# Smoking and the risk of type 2 diabetes in Japan: A systematic review and meta-analysis

**DOI:** 10.1016/j.je.2016.12.017

**Published:** 2017-07-14

**Authors:** Shamima Akter, Atsushi Goto, Tetsuya Mizoue

**Affiliations:** a Department of Epidemiology and Prevention, Center for Clinical Sciences, National Center for Global Health and Medicine, Tokyo, Japan; b Epidemiology and Prevention Group, Center for Public Health Sciences, National Cancer Center, Tokyo, Japan

**Keywords:** Smoking, Smoking cessation, Japanese, Type 2 diabetes, Meta-analysis, Systematic review

## Abstract

Cigarette smoking is the leading avoidable cause of disease burden. Observational studies have suggested an association between smoking and risk of type 2 diabetes mellitus (T2DM). We conducted a meta-analysis of prospective observational studies to investigate the association of smoking status, smoking intensity, and smoking cessation with the risk of T2DM in Japan, where the prevalence of smoking has been decreasing but remains high. We systematically searched MEDLINE and the Ichushi database to December 2015 and identified 22 eligible articles, representing 343,573 subjects and 16,383 patients with T2DM. We estimated pooled relative risks (RRs) using a random-effects model and conducted subgroup analyses by participant and study characteristics. Compared with nonsmoking, the pooled RR of T2DM was 1.38 (95% confidence interval [CI], 1.28–1.49) for current smoking (19 studies) and 1.19 (95% CI, 1.09–1.31) for former smoking (15 studies). These associations persisted in all subgroup and sensitivity analyses. We found a linear dose-response relationship between cigarette consumption and T2DM risk; the risk of T2DM increased by 16% for each increment of 10 cigarettes smoked per day. The risk of T2DM remained high among those who quit during the preceding 5 years but decreased steadily with increasing duration of cessation, reaching a risk level comparable to that of never smokers after 10 years of smoking cessation. We estimated that 18.8% of T2DM cases in men and 5.4% of T2DM cases in women were attributable to smoking. The present findings suggest that cigarette smoking is associated with an increased risk of T2DM, so tobacco control programs to reduce smoking could have a substantial effect to decrease the burden of T2DM in Japan.

## Highlights

• This meta-analysis examined smoking and diabetes risk among Japanese.
• Current and former smokers showed a higher risk of diabetes than non-smokers.
• Diabetes risk linearly increased with higher consumption of cigarettes.
• Diabetes risk steadily decreased after smoking cessation.

## Introduction

The United States Surgeon General's report recently documented a 40% increase in the risk of type 2 diabetes mellitus (T2DM) among cigarette smokers compared with nonsmokers, based on a systematic review and meta-analysis of 46 prospective studies, and concluded that cigarette smoking is a cause of T2DM.^1^ This conclusion has been supported by a more recent and vigorous systematic review and meta-analysis of 88 prospective studies.^2^ Although it remains debatable whether a causal relationship between smoking and T2DM has been established,^2^, ^3^, ^4^ eliminating smoking may considerably reduce the burden of T2DM. For example, Pan et al estimated that 11.7% of diabetes cases among men and 2.4% of diabetes cases among women would be attributable to active smoking if smoking is causally related to diabetes.^2^ Because there are substantial differences in the prevalence of smoking among countries,^5^ the burden of diabetes that is attributable to smoking likely varies across countries. Quantification of the country-specific burden of diabetes associated with smoking would help guide country-specific evidence-based policies.

In Japan, the prevalence of diabetes has been steadily increasing and is expected to increase 10% by 2030.^6^ Obesity is not common in Japan,^7^ so preventative strategies that target weight loss may not be as effective in Japan as in Western populations.^8^ Given the high prevalence of smoking, especially among young men (approximately 32%),^7^ tobacco control may have a substantial importance in managing diabetes in Japan. However, there has been no systematic evaluation of the association between smoking and the risk or burden of diabetes in Japan. Recent systematic reviews^1^, ^2^ of worldwide studies did not include two Japanese studies.^9^, ^10^ Furthermore, increasing evidence from epidemiological studies also suggests that passive smoking is associated with an increased risk of diabetes.^2^, ^11^, ^12^, ^13^ Therefore, the present study was performed to provide 1) a quantitative summary of the association between smoking status (current smoking, former smoking, smoking cessation years, and passive smoking) and the risk of T2DM in Japan and 2) the population attributable fraction (PAF) of diabetes due to smoking in Japan.

## Methods

### Search strategy

We conducted a systematic search of MEDLINE for the literature published through December 2015 of studies addressing the association between tobacco smoking and T2DM. The Ichushi (Japana Centra Revuo Medicina) database was also searched to identify studies written in Japanese. We used the following texts and keywords in combination with both MeSH terms and text words: diabetes mellitus, type 2 or diabetes mellitus, prediabetic state, smoking, smoking cessation, passive smoking, tobacco, smokeless tobacco use, cigarette, incidence, cohort studies, follow-up studies, survival analysis, Japan, and Japanese. We also searched the reference lists of publications included in the meta-analysis and relevant reviews.

### Selection criteria and data extraction

We identified articles eligible for further review by performing an initial screen of identified abstracts or titles. The second screening was based on the full-text review. Two investigators (SA and AG) independently assessed the full text for eligibility; discrepancies were resolved via consensus or determined by a third investigator (TM). Only prospective cohort studies of Japanese populations living in Japan were included. We also considered studies for inclusion if the investigators reported data from an original study and the study was conducted among adults without T2DM at baseline. Exclusion criteria were studies that included participants with a specific disease. In case of multiple publications related to the same study, we included the reports with the longest follow-up or the largest number of incident cases of T2DM.

From full-text articles, we extracted data on the year of publication, study design, number of participants, exposures, the time of the exposures assessment, outcomes, confounders, and the measures of association. The main exposure variable of interest was the presence or absence of tobacco smoking at baseline. The preferred reference group was never smokers. The majority of studies defined a group of former smokers, but a few studies defined smokers and nonsmokers without mentioning whether former smokers were included in the nonsmoking group.

The outcome variable of interest was T2DM. The definitions and diagnostic criteria to define T2DM varied somewhat across studies. The criteria used to define T2DM have changed over time, as is evident by comparing the World Health Organization 1985 criteria^14^ (fasting plasma glucose [FPG] ≥140 mg/dL) with the World Health Organization 1999 criteria^15^ or the American Diabetes Association 1997 criteria^16^ (FPG ≥126 mg/dL). Some recent studies published after 2010 also used hemoglobin A1c (HbA1c) in defining T2DM based on American Diabetes Association criteria^17^ (FPG ≥126 mg/dL or HbA1c ≥ 6.5%). The diagnosis of diabetes was based on objective measurement (blood tests) except for in one study, which solely based diagnosis on self-reporting by patients. We included information available from publications, but when we did not obtain sufficient information about the outcome, exposure, and study design from the article, we communicated with the authors of the original reports for further details.

### Quality assessment of the included studies

Using the Newcastle-Ottawa Scale,^18^ we assessed the overall quality of each study by totaling scores of the 9 criteria (0–9 stars): the representativeness of the exposed cohort, the selection of the nonexposed cohort, ascertainment of exposure, and outcome of interest not present at the start of the study (maximum of 4 stars); comparability of the cohorts on the basis of study design and analysis (maximum of 2 stars); and finally, the assessment of the outcome (maximum of 3 stars). Studies with scores of ≥6, 4–5, and 0–3 were defined as a high, moderate, and low quality studies, respectively.

### Statistical analysis

Relative risks (RRs) were used as the common measure of association across studies. Hazard ratios and incidence density ratios were directly considered as RRs, and odds ratios were regarded as approximate to RRs in view of the low incidence rates. Pooled risk estimates were performed according to the type of smoking. We used DerSimonian and Laird random-effects models for calculating the summary estimates.^19^ We used funnel plots and Egger's regression asymmetry test to assess publication bias.^20^ Additionally, we performed trim-and-fill procedures to further evaluate possible effects of publication bias.^21^ We also conducted subgroup analyses according to follow-up years (≤10 vs. >10 years), sample size (≤20,000 vs. >20,000), number of confounding factors (≤8 vs. >8 factors), mean age (≤50 vs. >50 years), and diagnostic criteria of diabetes (FPG≥126 mg/dL only vs. FPG≥126 mg/dL or HbA1c ≥ 6.5). We assessed the difference in association between groups using meta-regression analysis. We undertook sensitivity analyses by excluding studies in which former smokers were included in the nonsmoker group.

In assessing dose-response relationships, we treated the number of cigarettes smoked per day as the explanatory variable. Because most studies reported cigarette consumption as categorical data, we assigned the mid-value of each category and 0 to nonsmokers (reference). For the highest open-ended category, the assigned number of cigarettes smoked per day was calculated as the lower boundary multiplied by 1.2.^22^ We used a random-effects generalized least-squares regression model to assess the pooled dose-response relation between smoking and risk of T2DM. To examine the potential nonlinear relationship of the number of cigarettes consumed with the risk of T2DM, we used restricted cubic splines with three knots placed at the 10th, 50th, and 90th percentiles of the distribution. We assessed the effects of smoking cessation on the risk of T2DM from 3 studies^9^, ^23^, ^24^ that reported the risk of T2DM in relation to duration of smoking cessation. We considered three categories of smoking cessation: less than 5 years, 5–9 years, and 10 years or more. Never smoking was used as reference category in all the studies. One study^23^ presented results for two separate categories of less than 5 years since quitting smoking (<3 and 3–5 years) and another study^24^ presented results for three separate categories of 10 years or more since quitting smoking (10 to <15, 15 to <20, and ≥20 years). We combined those additional categories using our meta-analysis approach.

We estimated the PAF using the formula [prevalence of smoking × (RR−1)/{prevalence of smoking × (RR−1) + 1}], where RR indicates pooled RRs. The national prevalence of past and current smokers among adults (≥20 years of age)^25^ was used to estimate the PAF. We used Stata version 13.1 (StataCorp, College Station, TX, USA) for all analyses.

## Results

### Study selection

**Fig. 1:**
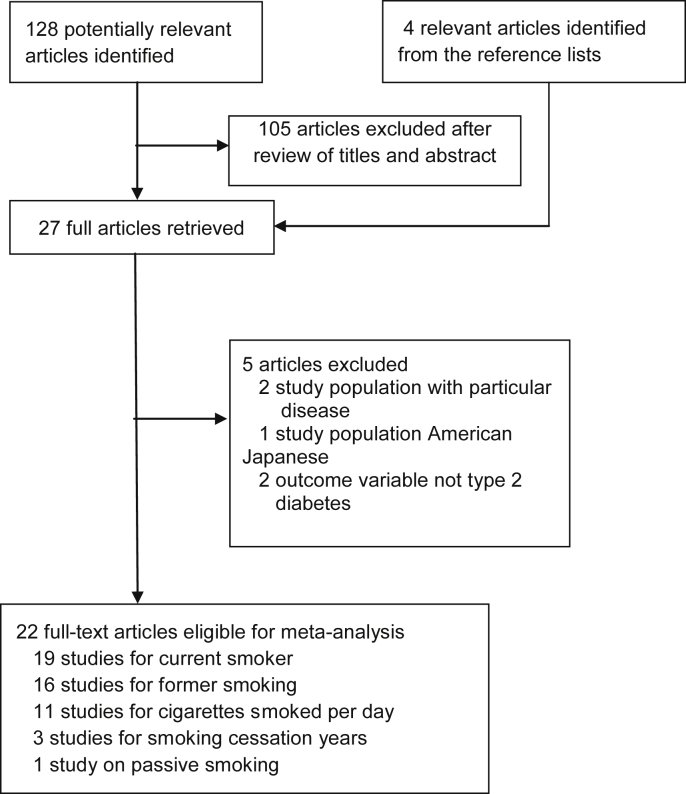
Flowchart of the selection of studies included in meta-analysis.

Our initial search identified 128 potential articles, of which 23 articles were considered potentially eligible based on the title and abstract screening (Fig. 1). Another four articles were identified from reference lists. A total of 27 full-text articles were reviewed. Of these, five articles met exclusion criteria, leaving 22 articles (19 studies) in our meta-analysis. Of these, 19 articles focused on current smoking, of which 16 articles also included results for former smoking. Of two articles based on a single study,^24^, ^26^ one^24^ assessed the association with current and former smoking, whereas the other^26^ assessed the association with smoking intensity. Of two articles from another study,^23^, ^27^ one assessed the association with current and former smoking,^27^ whereas the other^23^ assessed the association with smoking cessation years. We found only one article investigating the association between passive smoking and risk of diabetes.^28^

### Study characteristics

A total of 19 independent prospective cohort studies including 343,573 individuals and 16,383 incident cases were identified (Table 1).^9^, ^10^, ^24^, ^27^, ^28^, ^29^, ^30^, ^31^, ^32^, ^33^, ^34^, ^35^, ^36^, ^37^, ^38^, ^39^, ^40^, ^41^, ^42^ The selected studies were published between 1997 and 2015. The number of subjects per study ranged from 1266 to 128,141. The average follow-up duration ranged from 4 to 16 years. Diabetes was ascertained using biological screening in all studies except one (patient report).^24^ Smoking status was self-reported in all studies. Six studies involved men only,^35^, ^36^, ^37^, ^39^, ^41^, ^42^ and the remaining studies involved both men and women. Regarding cutoff points for diabetes, 11 studies used a FPG threshold of ≥126 mg/dL,^10^, ^28^, ^30^, ^32^, ^33^, ^34^, ^36^, ^37^, ^38^, ^39^, ^42^ three studies used FPG ≥126 mg/dL or HbA1c ≥ 6.5%,^9^, ^27^, ^31^ one study used FPG ≥140 mg/dL,^35^ one study used FPG ≥110 mg/dL,^40^ one study used FPG ≥126 mg/dL or 2-h post-load glucose ≥200 mg/dL,^29^ and one study used HbA1c ≥ 6.1%.^41^ Most studies adjusted for age (19 studies), body mass index (18 studies), alcohol consumption (13 studies), physical activity (11 studies), and heredity (9 studies), whereas fewer were adjusted for education (1 study), diet (1 study), and waist circumference (1 study) (eTable 1). The characteristics of studies on the number of years of smoking cessation^9^, ^24^, ^27^ are shown in eTable 2. All the studies included in the present meta-analysis were generally of high quality (eTable 3).

**Table 1:** Characteristics of studies included in the meta-analysis.

Source	Study designation	Sex	Baseline age group	Sample size	Maximum follow-up years	Number of cases	Diabetes incidence by smoking status, Number/Total			Diabetes ascertainment	Diabetes detection by FPG (mg/dL) or HbA1c	Baseline diabetes ascertainment
							Current	Non	Former			
Kawakami et al. 1997	Japanese Cohort of Male Employees	Men	18–53	2312	8	41	317/1420	147/583	41/309	Biological screening	FPG≥140	Patient questionnaire
Sugimori et al. 1998	Database accumulated from MHTS	Men and Women	18–69	2573	16	296	NA	NA	NA	Biological screening	FPG≥110	Biological screening
Uchimoto et al. 1999	Osaka Health Survey	Men	35–60	6250	16	450	302/3880	69/1068	79/1302	Biological screening	FPG≥126	Biological screening
Nakanishi et al. 2000	Japanese male office workers	Men	35–59	1266	5	54	42/646	7/407	5/213	Biological screening	FPG≥126	Biological screening
Sawada et al. 2003	Male employees	Men	20–40	4745	14	280	195/3190	82/1555	NA	Biological screening	FPG≥126	Biological screening
Sairenchi et al. 2004	Japanese subjects who underwent health checkup	Men and Women	40–79	128,141	9	7990	2027/NA	4815/NA	1148/NA	Biological screening	FPG≥126	Biological screening
Hayashino et al. 2008	HIPOP-OHP study	Men and women	mean age 38.2	6498	4	229	NA/2900	NA/2129	NA/779	Biological screening	FPG≥126	Biological screening
Nagaya et al. 2008	Gifu Prefectural Center for Health Check and Health Promotion study	Men	30–59	16,829	11	869	445/9807	193/3882	213/4140	Biological screening	FPG≥126	Biological screening
Fukui et al. 2011	Annual health examination at Sakazaki Clinic in Kyoto	Men and women	Mean age 48.2	5152	11	262	670/NA	3077/NA	557/NA	Biological screening	FPG≥126	Biological screening
Ide et al. 2011	Civil service officers undergoing annual health checkup	Men and Women	30–59	5848	7	287	NA	NA	NA	Biological screening	FPG≥126	Biological screening
Morimoto et al. 2012	Japanese individuals undergoing health check-up at central hospital in Nagoya	Men and Women	40–69	5872	16	246	119/1043	377/4114	99/715	Biological screening	FPG≥126 or HbA1c ≥ 6.5	Biological screening
Teratani et al. 2012	Workers at a Japanese steel company	Men	mean age 40	8423	8	464	275/4761	189/3662	NA	Biological screening	HbA1c ≥ 6.1	Biological screening
Heianza et al. 2012	TOPICS 6	Men and women	40–75	7654	5	289	NA	NA	NA	Biological screening	FPG≥126 or HbA1c ≥ 6.5	Biological screening
Katsuta et al. 2012	Urban residents of Osaka city	Men and women	40–74	9273	4	166	114/7459	11/239	41/1519	Biological screening	FPG≥126	Biological screening
Doi et al. 2012	Suburban residents of Hisayama city, Kyushu	Men and women		1935	14	286	NA	NA	NA	Biological screening	FPG≥126 or 2-h post-load glucose≥200	Biological screening
Oba et al. 2012	JPHC Study	Men and women	40–59	59834	10	1100	340/13136	548/38131	144/6325	Patient report	NA	Patient questionnaire
Kaneto et al. 2013	MY Health UP Study	Men and women	36–55	13,700	5	408	146/4795	194/7262	68/1643	Biological screening	FPG≥126	Biological screening
Hilawe et al. 2015	Aichi workers cohort study	Men and Women	35–66	3338	9	225	85/954	75/1608	65/776	Biological screening and patient questionnaire	FPG≥126	Biological screening and patient questionnaire
Akter et al. 2015	J-ECOH study	Men and Women	15–83	53,930	4	2441	1074/20579	568/10162	799/23189	Biological screening	FPG≥126 or HbA1c ≥ 6.5	Biological screening

FPG, fasting plasma glucose; HbA1c, glycated hemoglobin A1c; NA, not available.

### Smoking and risk of diabetes

**Fig. 2:**
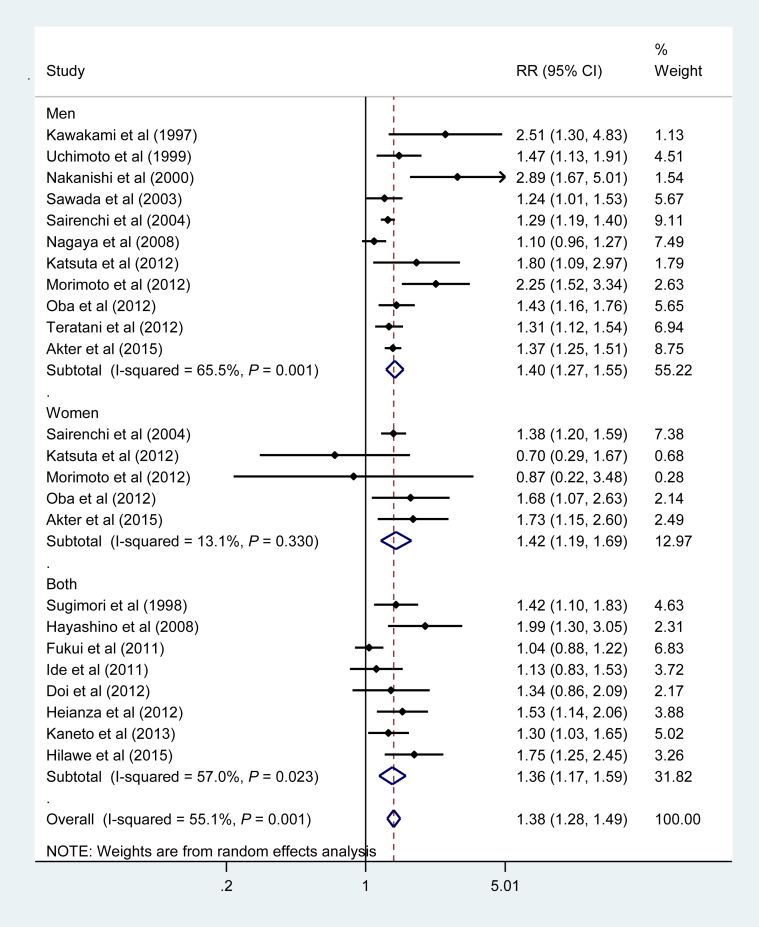
Adjusted relative risk for current smokers compared with non-smokers. CI, confidence interval; RR, relative risk.

Fig. 2 shows the pooled RR for the association between active smoking and risk of T2DM. Active smokers had an increased risk of T2DM compared with nonsmokers, with a pooled RR of 1.38 (95% confidence interval [CI], 1.28–1.49). The RR was virtually the same for men and women: 1.40 (95% CI, 1.27–1.55) for men and 1.42 (95% CI, 1.19–1.69) for women. There was evidence of statistical heterogeneity of RRs across studies for the overall study population (I^2^ = 55.1%, *P* = 0.001) and for men only (I^2^ = 65.5%, *P* = 0.001). Among 15 studies^9^, ^10^, ^24^, ^27^, ^28^, ^30^, ^32^, ^33^, ^34^, ^35^, ^36^, ^37^, ^38^, ^41^, ^42^ that used never smokers (without former smokers) as the reference, the pooled RR was 1.39 (95% CI, 1.28–1.52).

### Stratified analysis

To examine sources of heterogeneity on the association between active smoking and T2DM, we conducted stratified analysis across a number of key study characteristics (Table 2). An increased risk of diabetes in current smokers was found in most of the subgroups. A slightly stronger association between smoking and T2DM was found in studies that adjusted for more than eight confounding factors, with a mean follow-up period of ≤10 years, and when diabetes was diagnosed using both FPG and HbA1c. However, we found no significant differences across strata (the *P* values for meta-regression were >0.05 for all).

**Table 2:** Stratified analysis of pooled relative risks of diabetes for current smokers.

Stratified analysis	Number of studies	Polled RR (95% CI)	*P* value	
			Heterogeneity	Meta-regression
**Sex**^a^				
Men	12	1.40 (1.27–1.55)	0.002	0.91
Women	5	1.42 (1.19–1.69)	0.33	
**Maximum follow-up, years**				
≤10 years	12	1.41 (1.29–1.53)	0.05	0.06
>10 years	7	1.24 (1.09–1.40)	0.08	
**Mean age, years**				
≤50 years	13	1.34 (1.21–1.48)	0.001	0.41
>50 years	6	1.37 (1.25–1.49)	0.37	
**Sample size**				
≤20000	16	1.38 (1.24–1.54)	<0.01	0.96
>20000	3	1.33 (1.26–1.41)	0.53	
**Adjustment for confounding factors**				
≤8 factors	7	1.30 (1.17–1.46)	0.07	0.52
>8 factors	12	1.39 (1.25–1.55)	0.005	
**Diagnostic criteria of diabetes**^b^				
FPG ≥126 mg/dL	11	1.32 (1.19–1.47)	0.001	0.22
FPG ≥126 mg/dL or HbA1c ≥6.5	3	1.46 (1.28–1.66)	0.14	

CI, confidence interval; FPG, fasting plasma glucose; HbA1c, glycated hemoglobin A1c; RR, relative risk.

a In two studies results were reported only for both men and women.

b In 5 studies diabetes were diagnosed as FPG ≥140, FPG ≥110, HbA1c ≥ 6.1, FPG ≥126 or 2-h post-load glucose ≥200, and self-report.

### Publication bias

Visual inspection of a funnel plot indicated asymmetry in studies related to T2DM, which raises the possibility of publication bias (eFigure 1A). We then performed sensitivity analysis using a trim-and-fill method (eFigure 1B), which hypothetically imputes six negative and unpublished studies that were missing from the initial analysis. The imputed studies produced a symmetrical funnel plot. The pooled analysis including the six hypothetical studies also showed a statistically significant association between active smoking and T2DM (RR 1.27; 95% CI, 1.16–1.38).

### Former smoking and risk of diabetes

**Fig. 3:**
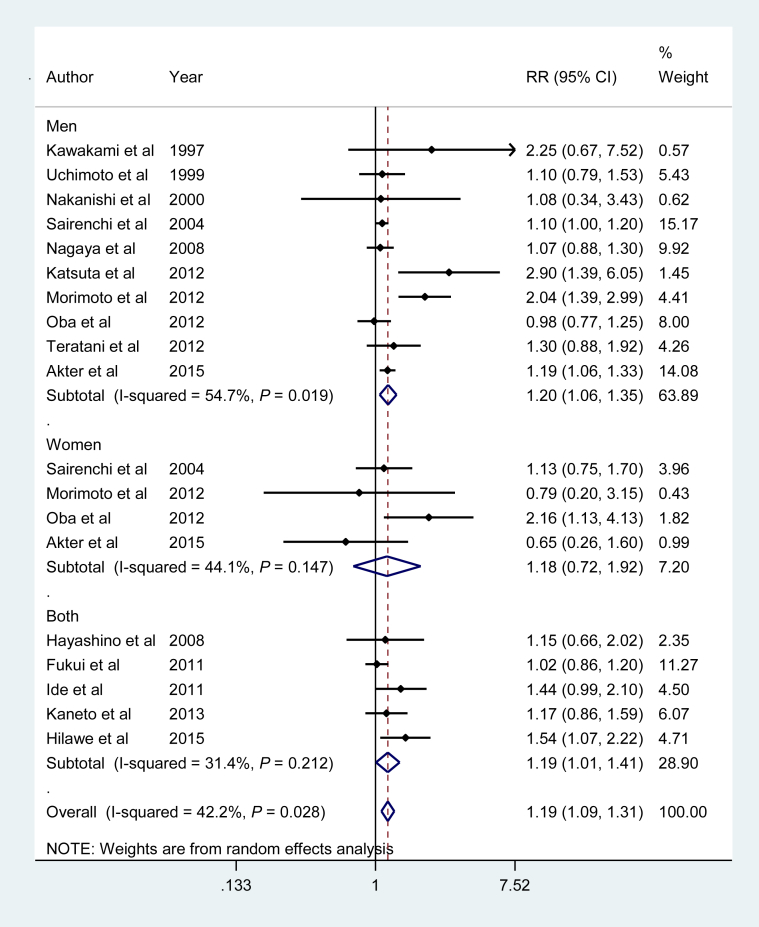
Adjusted relative risk for past smokers compared with nonsmokers. CI, confidence interval; RR, relative risk.

Fig. 3 shows the pooled RR for the association between former smoking and risk of T2DM. Former smokers had an increased risk of T2DM compared with nonsmokers, with a pooled RR of 1.19 (95% CI, 1.09–1.31), which did not differ by sex: RRs were 1.20 (95% CI, 1.06–1.35) and 1.18 (95% CI, 0.72–1.92) for men and women, respectively. There was evidence of statistical heterogeneity of RRs across studies for the overall population (I^2^ = 42.2%, *P* = 0.03) and for men only (I^2^ = 54.7%, *P* = 0.02).

### Dose-response relationship between smoking, smoking cessation years, and T2DM

**Fig. 4:**
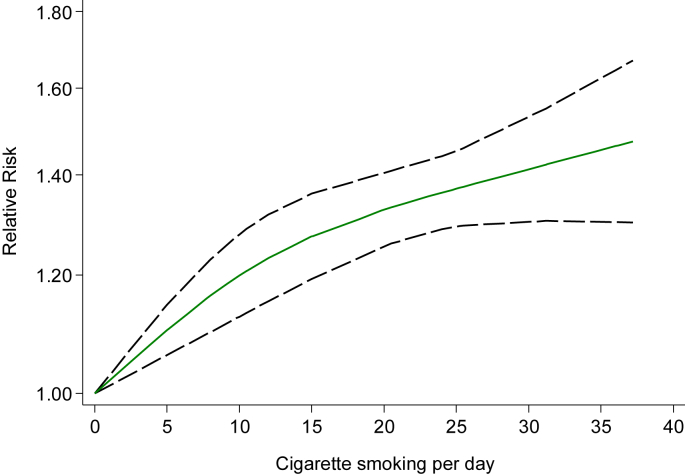
Linear dose-response relationship between cigarette smokes per day and relative risk of diabetes among total subjects (P for non-linearity = 0.08). Data were modeled with random-effects restricted cubic spline models with three knots placed at 10th, 50th, and 90th percentiles of cigarette smokes per day. Lines with long dashes represent the pointwise 95% confidence intervals for the fitted linear trend (solid line). Line with short dashes represents the linear trend.

**Fig. 5:**
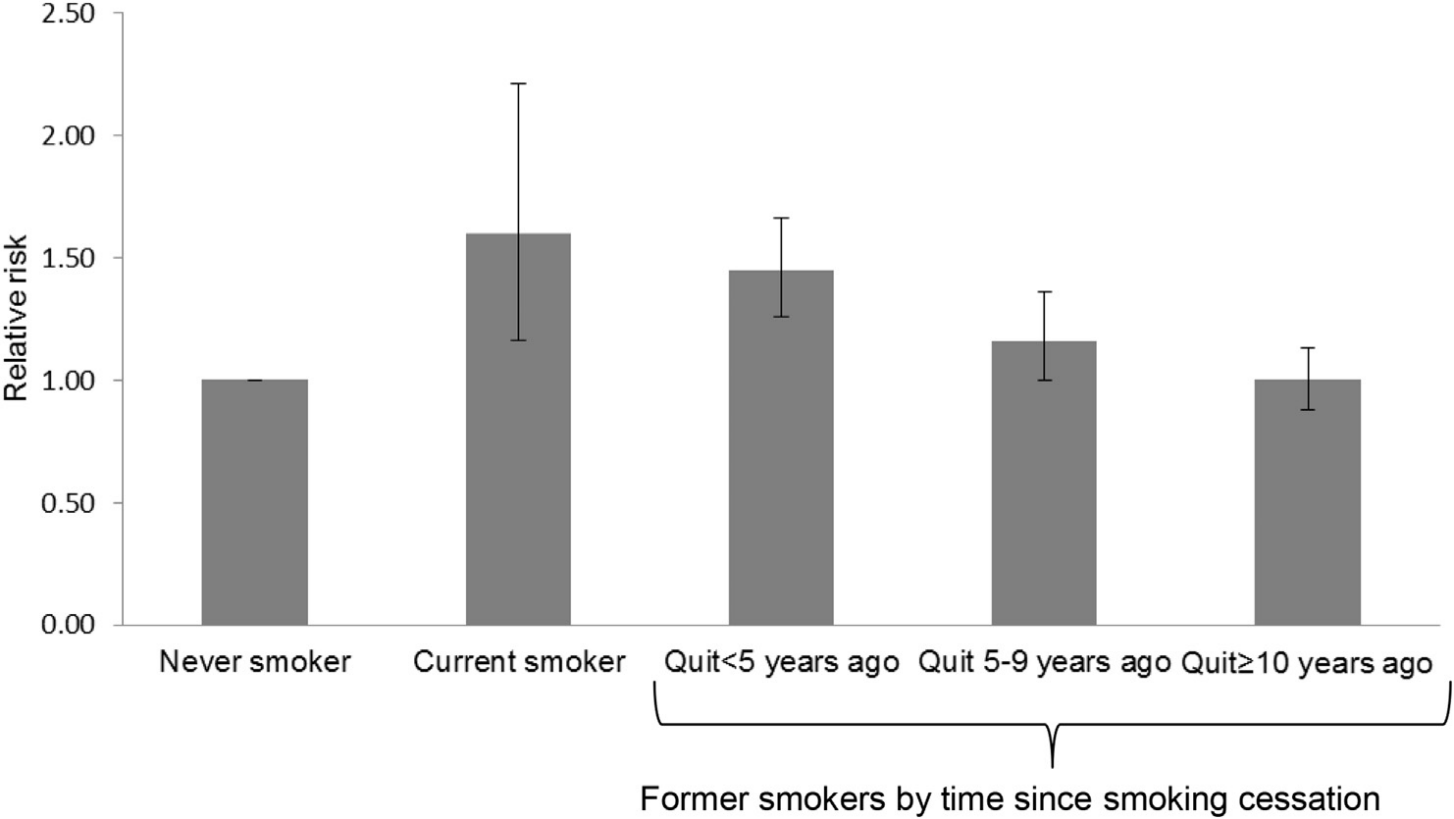
Relationship between duration of smoking cessation and relative risk of diabetes. Data were pooled using random-effects meta-analysis from three studies that presented data for duration of smoking cessation. Error bars show 95% confidence intervals.

Among 12 studies^9^, ^10^, ^26^, ^29^, ^32^, ^35^, ^36^, ^37^, ^38^, ^39^, ^41^, ^42^ that reported the association between the amount of cigarette consumption and incidence of T2DM, we evaluated dose-response relationships (Fig. 4). We observed a linear increase in T2DM risk with increasing cigarette consumption (*P* for nonlinearity = 0.08), with the risk of T2DM being increased by 16% for each increment of 10 cigarettes per day. We assessed the effects of duration of smoking cessation on the risk of T2DM from 3 studies^9^, ^23^, ^24^ (Fig. 5). As compared to never smokers, the pooled RRs of T2DM was 1.60 (95% CI, 1.16–2.21) for current smokers, 1.45 (95% CI, 1.26–1.66) for new quitters (<5 years), 1.16 (95% CI, 1.00–1.36) for middle-term quitters (5–9 years), and 1.00 (95% CI, 0.88–1.13) for long-term quitters (≥10 years).

### PAF estimations

Using the national prevalence of current smokers (total, 20.7%; men, 34.1%; women, 9.0%) and former smokers (total, 22.2%; men 36.2%; women 10.0%) among adults ≥20 years of age in Japan^25^ and the summary RR obtained from all studies, the PAF of T2DM due to current smoking was 7.3% (95% CI, 5.5–9.2%) for the total population, 12.0% (95% CI, 8.4–15.8%) for men, and 3.6% (95% CI, 1.7–5.8%) for women, and that due to former smoking was 4.1% (95% CI, 2.0–6.4%), 6.8% (95% CI, 2.1–12.1%) and 1.8% (95% CI, −2.9 to 8.4%), respectively.

### Passive smoking and risk of diabetes

Exposure to passive smoking, excluding active smoking, was associated with an increased risk of diabetes compared with no current exposure to passive smoking,^28^ although the association was not statistically significant, with a RR of 1.20 (95% CI, 0.54–2.68) (eTable 4).

## Discussion

In this systematic review and meta-analysis involving 343,573 subjects and 16,383 patients with T2DM from 19 prospective cohort studies in Japan, we found an increased risk of T2DM in smokers compared with nonsmokers. An increased risk associated with current smoking was observed in most of the subgroups. There was a dose-response relationship among smokers, with the risk of T2DM being increased by 16% for each increment of 10 cigarettes smoked per day. The risk of T2DM remains high among those who quit during the preceding 5 years but decreased steadily with increasing duration of cessation, reaching a risk level comparable to that of never smokers after 10 years of smoking cessation.

We found a significant 38% higher risk of T2DM for current smoking compared with nonsmoking among the Japanese. The magnitude of this association is consistent with three meta-analyses conducted so far on active smoking and T2DM worldwide.^1^, ^2^, ^43^ In previous meta-analyses, Pan et al found a 35% higher risk,^2^ the United States Surgeon General's report found a 37% higher risk,^1^ and Willi et al found a 44% higher risk^43^ of T2DM in current smokers compared with current nonsmokers. The present meta-analysis of Japanese studies additionally included two large studies (1 recent study^9^ and another study written in Japanese^10^) that were not included in previous meta-analyses. We found a dose-response relationship between smoking and T2DM, a finding that is also consistent with previous studies.^1^, ^2^, ^43^

The association between smoking and T2DM is biologically plausible. Smoking leads to insulin resistance or inadequate compensatory insulin secretion^44^, ^45^ through various underlying effects, including oxidative stress, inflammation, and endothelial dysfunction.^46^, ^47^ Nicotine in cigarettes may also exert a direct toxic effect on beta-cell function.^48^ In addition, although smoking tends to decrease weight, it leads to central adiposity,^49^, ^50^ which has been linked to inflammation^51^ and insulin resistance.^52^

Compared with nonsmoking, former smoking was associated with a 19% higher risk of T2DM in the present study. This estimate is similar to those of two other recent meta-analyses,^1^, ^2^ where former smoking was associated with a 14% higher risk of diabetes. In a meta-analysis, Pan et al reported that former smoking was associated with a 16% higher risk of diabetes in Asians and a 15% higher risk of diabetes in East Asians.^2^ That study included data of 13 Japanese studies, but our study included two additional studies.^9^, ^10^ When we examined the association between the duration of quitting smoking and T2DM, we found a significantly increased risk of T2DM for the first 5 years of smoking cessation compared with nonsmoking, although this increase did not exceed the risk of T2DM among current smokers. The risk of T2DM decreased steadily with increasing duration of cessation, reaching a risk level comparable to that of never smokers after 10 years of smoking cessation. Consistent with our finding, two recent meta-analyses reported that smoking cessation was associated with a substantial decrease in diabetes risk in the long term.^2^, ^53^ Taken together, although the risk of T2DM remains high after short-term smoking cessation, it decreases eventually in the long run.

As smoking cessation usually leads to weight gain,^54^ a concern has been raised about the possibility of increased risk of T2DM after quitting smoking. In fact, mechanistic studies showed deterioration of insulin sensitivity and lipid profiles after smoking cessation.^55^, ^56^ Some studies in Japan,^24^ Korea,^57^ and the United States^58^, ^59^ have reported a sizable increase (>15%) in the risk of diabetes among new quitters compared with current smokers. However, we did not find any further increase in risk after smoking cessation. Given the limited number of studies with conflicting data, further studies are required to elucidate whether short-term smoking cessation could lead to an increased risk of T2DM after quitting smoking.

So far, four meta-analyses based on worldwide data reported that passive smoking is associated with a 21–28% higher risk of T2DM.^2^, ^11^, ^12^, ^13^ We found only one prospective study^28^ on the association between passive smoking and T2DM in Japan, which reported a nonsignificant 20% higher risk of T2DM associated with passive smoking (eTable 4). In that study, exposure to passive smoke in the workplace was associated with an increased risk of diabetes (hazard ratio 1.81; 95% CI, 1.06–3.08). In Japan, the Health Promotion Act was enacted in 2003 to restrict exposure to secondhand smoke in the workplace.^60^ According to this act, smoking is banned in public spaces, relegating smokers to designated areas. The law was strengthened in 2010.^60^ Accordingly, passive smoking is expected to decrease in Japan, so the impact of passive smoking on diabetes might have been decreasing.

The prevalence of smoking has been declining in Japan; however, smoking remains a public health threat, as 20.7% of adults smoke.^25^ We estimated that 12.0% of T2DM cases in men and 3.6% in women were attributable to current smoking in Japan. For former smoking, these figures were 6.8% and 1.8% in men and women, respectively. A recent meta-analysis reported that worldwide, 11.7% of T2DM cases in men and 2.4% in women were attributable to current smoking,^2^ but the PAF for former smoking was not reported. The present findings suggest that active and former smoking jointly accounted for nearly one-fifth of T2DM cases in Japanese men (18.8%). Smoking prevention should be encouraged and effective smoking control programs should be implemented to reduce smoking-related diabetes in Japan.

The strengths of our study include being based on high-quality cohort studies with large sample sizes and the inclusion of all relevant studies among Japanese populations, covering recent well-designed cohort studies. This enabled us to draw strong conclusions. Despite these strengths, some limitations of this meta-analysis must be considered. First, there was heterogeneity in the RRs across studies that might result from differences in participant characteristics and definitions of outcome measures. However, we conducted stratified analyses and found summary RRs consistently greater than 1 across a number of study- and participant-level characteristics. Second, funnel plot analysis showed some asymmetry among male smokers, suggesting the possibility of publication bias and missing of some gray literature. We used a trim-and-fill method, which can capture all unpublished and gray literature, and obtained a similar result, suggesting that the association was not affected by unpublished negative studies. Third, the possibility of residual confounding and unmeasured factors cannot be ruled out in observational studies. Specifically, smoking is related to other unhealthy lifestyle factors, such as unhealthy diet, excessive alcohol use, physical inactivity, and comorbidities.^61^ We confirmed, however, that there were no substantial differences in association between studies with adjustment of ≥8 confounding variables and those with adjustment of fewer variables.

In conclusion, the results of this meta-analysis suggest that current smoking is associated with an increased risk of T2DM in a dose-dependent manner among the Japanese. Although the risk of diabetes remains high after short-term smoking cessation, the risk decreases substantially in the long run. Tobacco smoking accounts for 18.8% of T2DM cases among men and 5.4% of T2DM cases among women in Japan. These findings greatly strengthen the evidence on the association between smoking and T2DM and provide an additional rationale for the intensified implementation of tobacco control programs, especially in countries, like Japan, where tobacco smoking is still prevalent.

## Conflicts of interest

None declared.
