# Supplementary material for: Smoking and the risk of type 2 diabetes in Japan: A systematic review and meta-analysis
Source: J Epidemiol. 2017 Jul 14;27(12):553–61. doi: 10.1016/j.je.2016.12.017 (PMC5623034; doi:10.1016/j.je.2016.12.017)
Supplement: Supplementary file 1 [file mmc1.pdf]

eTable 1. Methods for analysis and confounding variables for smoking (current or former) and risk of type 2 diabetes

Source	Method for Analysis	Risk Estimates	Confounding Variables
Kawakami et al. 1997	Proportional hazards regression	HR	Age, BMI, heredity, physical activity, alcohol consumption, education, occupation, shift work
Sugimori et al. 1998	Proportional hazards regression	HR	Age, BMI, heredity, physical activity, blood pressures, total cholesterol, HDL cholesterol, triglyceride, glucose, eating breakfast, uric acid, dairy intake
Uchimoto et al. 1999	Proportional hazards regression	RR	Age, BMI, physical activity, heredity, alcohol consumption, fasting plasma glucose, total cholesterol, triglycerides, HDL cholesterol, hematocrit
Nakanishi et al. 2000	Proportional hazards regression	RR	Age, BMI, heredity, blood pressure, physical activity, alcohol consumption, total cholesterol, HDL cholesterol, triglyceride, uric acid, fasting glucose status, hematocrit
Sawada et al. 2003	Proportional hazards regression	HR	Age, BMI, heredity, blood pressure, alcohol consumption
Sairenchi et al. 2004	Proportional hazards regression	RR	Age, BMI, heredity, blood pressure, alcohol consumption, total cholesterol, triglycerides, HDL cholesterol, antihypertensive treatment, fasting glucose status
Hayashino et al. 2008	Proportional hazards regression	HR	Age, sex, BMI, alcohol consumption, physical activity, hypertension, health promotion intervention, sweetened beverage, vegetable intake, not care about eating too much fat
Nagaya et al. 2008	Proportional hazards regression	HR	Age, alcohol consumption, exercise, education
Fukui et al. 2011	Proportional hazards regression	HR	Age, sex, BMI, blood pressure, fasting glucose status, total cholesterol, triglyceride, HDL-cholesterol, r-GTP, uric acid, creatinine, white blood cell count, hemoglobin, alcohol consumption, physical activity, family history of diabetes, and family history of hypertension
Ide et al. 2011	Proportional hazards regression	HR	Age, sex, BMI, triglyceride, hypertension, HDL cholesterol, and gamma-glutamyl transpeptidase
Morimoto et al. 2012	Proportional hazards regression	HR	Age, BMI, alcohol consumption, blood pressure, exercise, fasting status, total cholesterol, triglyceride, heredity, blood glucose level.
Teratani et al. 2012	Proportional hazards regression	HR	Age, BMI, alcohol consumption, arterial pressure, serum cholesterol, aspartate aminotransferase, creatinine, shift work, physical activity, uric acid
Heianza et al. 2012	Multiple logistic regression	OR	Age, sex, BMI, family history of diabetes, resting heart rate, hypertension, history of hyperlipidemia, fasting plasma glucose, HbA1c
Doi et al. 2012	Proportional hazards regression	HR	Age, sex, BMI, central obesity, physical activity, hypertension, FPG levels, family history of diabetes
Katsuta et al. 2012	Multiple logistic regression	OR	Age, sex, BMI, alcohol consumption, physical activity
Oba et al. 2012	Multiple logistic regression	OR	Age, BMI, heredity, hypertension, alcohol consumption, physical activity, weight change, study area
Kaneto et al. 2013	Proportional hazards regression	HR	Age, sex, BMI change, BMI at age 20 years, job type, family history of diabetes, hypertension, exercise, hours of sleep, alcohol consumption, coffee consumption,

			regular mealtimes, job stress
Hilawe et al. 2015	Proportional hazards regression	HR	Age, sex, BMI, heredity, physical activity, alcohol consumption, sugar and energy, sleep duration, arterial pressure, total cholesterol to HDL cholesterol ratio, HOMA2-IR, triglyceride, adiponectin, leptin, C-reactive protein
Akter et al. 2015	Proportional hazards regression	HR	Age, sex, BMI, worksite, waist circumference, hypertension

BMI, body mass index; FPG, fasting plasma glucose; HbA1c, glycated hemoglobin A1c; HDL, high-density lipoprotein; HOMA2-IR, Homeostatis Model Assessment of insulin resistant; HR, hazard ratio; OR, odds ratio; RR, relative risk.

eTable 2. Characteristics of studies included in the Meta-analysis of smoking cessation years and risk of type 2 diabetes

Source	Study designation	Sex	Baseline age group	Sample size	Maximum follow-up years	Number of cases	Adjusted variables	Years since quitting smoking, HR (95% confidence interval)
Morimoto et al. 2012	Japanese individuals undergoing health check-up at central hospital in Nagoya	Men	40–69	2070	16	246	Age, blood glucose, fasting, SBP, total cholesterol, triglycerides, alcohol consumption, exercise, family history of diabetes, BMI (time varying covariate	BMI<23: <3y, 1.91 (0.60-6.06); (3-5)y, 1.95 (0.62-6.17); (6-9) y, 0.59 (0.13-2.64), 2.22 (1.05-4.69). BMI≥23: <3y, 2.98 (1.38-6.27); (3-5)y, 2.49 (1.18-5.26); (6-9) y, 1.77 (0.75-4.17), 2.47 (1.45-4.21).
Oba et al. 2012	JPHC Study	Men	40–69	35,320	5	1162	Age, hypertension, alcohol intake, family history of diabetes, study area, leisure time physical activity, BMI	<5 y, 1.41 (1.10-1.81); (5-9)y, 1.12 (0.87-1.46); (10-14) y, 0.73 (0.53-1.00); (15-19)y, 0.87 (0.61-1.25); ≥20 y, 0.95 (0.69-1.29).
Akter et al. 2015	J-ECOH Study	Men and Women	15–83	38,557	4	1889	Age, sex, worksite, hypertension	<5 y, 1.36 (1.14-1.62); (5-9)y, 1.23 (1.01-1.51); ≥10 y,1.02 (0.85-1.23).

BMI, body mass index; HR, hazard ratio; JPHC, Japan Public Health Center-based; J-ECOH, Japan Epidemiology Collaboration on Occupational Health; SBP, systolic blood pressure.

eTable 3. Assessment of study quality

Author		Selection					Comparability			Outcome			Total score
	Year	1	2	3	4		5A	5B		6	7	8	
		Exposed cohort truly/somewhat representative	Nonexposed cohort drawn from the same community	Ascertainment of exposure	Outcome of interest not present at start		Cohorts controlled for age and sex	Cohorts controlled for other important factor(s)		Quality of outcome assessment	Follow-up long enough for outcomes to occur	Complete accounting for cohorts	
Kawakami et al	1997		*		*		*	*		*	*		6
Sugimori et al	1998	*	*		*		*	*		*	*		7
Uchimoto et al	1999		*		*		*	*		*	*		6
Nakanishi et al	2000		*		*		*	*		*	*	*	7
Sawada et al	2003		*		*		*	*		*	*		6
Sairenchi et al	2004	*	*		*		*	*		*	*		7
Hayashino et al	2008		*		*		*	*		*	*		6
Nagaya et al	2008		*		*		*	*		*	*		6
Fukui et al	2011		*		*		*	*		*	*		6
Ide et al	2011		*		*		*	*		*	*		6
Morimoto et al	2012		*		*		*	*		*	*		6
Heianza et al	2012		*		*		*	*		*	*		6
Katsuta et al	2012		*		*		*	*		*	*		6
Doi et al	2012		*		*		*	*		*	*		6
Oba et al	2012	*	*		*		*	*			*		6
Kaneto et al	2013		*		*		*	*		*	*		6
Teratani et al	2012		*		*		*	*		*	*		6
Hilawe et al	2015		*		*		*	*		*	*		6
Akter et al	2015	*	*		*		*	*		*	*		7

eTable 4. Characteristics of studies and summary findings examining passive smoking and risk of type 2 diabetes in Japan

Reference, Year	n	Male (%)	Mean baseline age	Mean follow-up years	Number of cases	Reference group	Passive smoking HR (95% CI)
Hayashino et al, 2008	6498	79. 1	38.2	3.4	229	Never smoker + no exposed to passive smoke	Exposed at workplace: 1.81 (1.06-3.19)<br>Exposed at home: 0.80 (0.46-1.40)<br>Pooled HR: 1. 20 (0. 54-2.68)

CI, confidence interval; HR, hazard ratio.

(A)

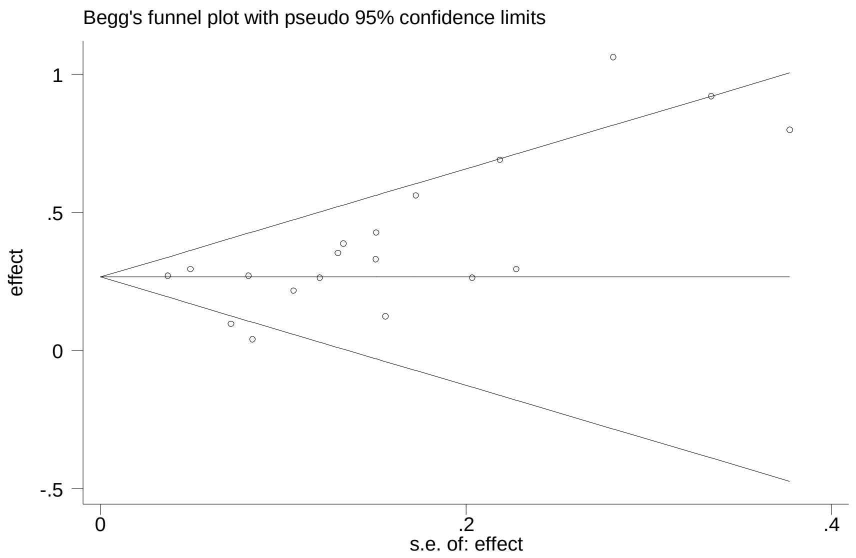

(B)

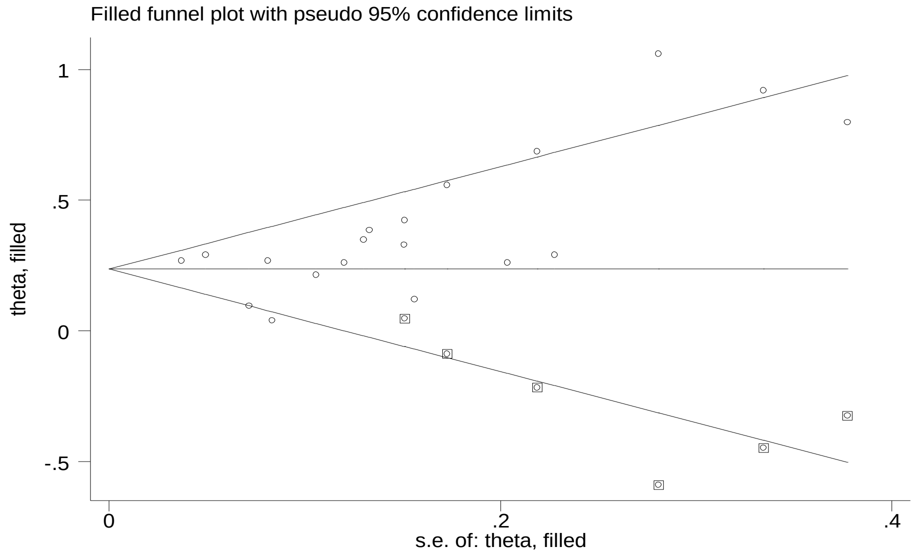

eFigure 1. Funnel plots without (A) and with trim and fill (B)
